# Have the recent advancements in cancer therapy and survival benefitted patients of all age groups across the Nordic countries? NORDCAN survival analyses 2002–2021

**DOI:** 10.2340/1651-226X.2024.35094

**Published:** 2024-04-10

**Authors:** Anna L.V. Johansson, Simon M. Kønig, Siri Larønningen, Gerda Engholm, Niels Kroman, Karri Seppä, Nea Malila, Bjarni Á. Steig, Eva María Gudmundsdóttir, Elínborg J. Ólafsdóttir, Frida E. Lundberg, Therese M.-L. Andersson, Paul C. Lambert, Mats Lambe, David Pettersson, Bjarte Aagnes, Søren Friis, Hans Storm

**Affiliations:** a Cancer Registry of Norway, Norwegian Institute of Public Health, Oslo, Norway; b Department of Medical Epidemiology and Biostatistics, Karolinska Institutet, Stockholm, Sweden; c Danish Cancer Institute, Danish Cancer Society, Copenhagen, Denmark; d Department Breast Surgery, Copenhagen University Hospital (Herlev/Gentofte), Copenhagen, Denmark; e Finnish Cancer Registry, Helsinki, Finland; f Faculty of Social Sciences, Tampere University, Tampere, Finland; g National Hospital of the Faroe Islands, Tórshavn, Faroe Islands; h Icelandic Cancer Registry, Reykjavík, Iceland; i Department of Oncology-Pathology, Karolinska Institutet, Stockholm, Sweden; j Biostatistics Research Group, Department of Health Sciences, University of Leicester, UK; k Regional Cancer Center Mid-Sweden, Uppsala, Sweden; l Swedish Cancer Registry, National Board of Health and Welfare, Stockholm, Sweden; m Danish Cancer Society, Copenhagen, Denmark

**Keywords:** cancer survival, epidemiology, Denmark, Finland, Iceland, Norway, Sweden

## Abstract

**Background:**

Since the early 2000s, overall and site-specific cancer survival have improved substantially in the Nordic countries. We evaluated whether the improvements have been similar across countries, major cancer types, and age groups.

**Material and methods:**

Using population-based data from the five Nordic cancer registries recorded in the NORDCAN database, we included a cohort of 1,525,854 men and 1,378,470 women diagnosed with cancer (except non-melanoma skin cancer) during 2002–2021, and followed for death until 2021. We estimated 5-year relative survival (RS) in 5-year calendar periods, and percentage points (pp) differences in 5-year RS from 2002–2006 until 2017–2021. Separate analyses were performed for eight cancer sites (i.e. colorectum, pancreas, lung, breast, cervix uteri, kidney, prostate, and melanoma of skin).

**Results:**

Five-year RS improved across nearly all cancer sites in all countries (except Iceland), with absolute differences across age groups ranging from 1 to 21 pp (all cancer sites), 2 to 20 pp (colorectum), -1 to 36 pp (pancreas), 2 to 28 pp (lung), 0 to 9 pp (breast), -11 to 26 pp (cervix uteri), 2 to 44 pp (kidney), -2 to 23 pp (prostate) and -3 to 30 pp (skin melanoma). The oldest patients (80–89 years) exhibited lower survival across all countries and sites, although with varying improvements over time.

**Interpretation:**

Nordic cancer patients have generally experienced substantial improvements in cancer survival during the last two decades, including major cancer sites and age groups. Although survival has improved over time, older patients remain at a lower cancer survival compared to younger patients.

## Introduction

In all Nordic countries, cancer survival has improved consistently since the early 2000s [1]. These improvements have been observed for both men and women, and across a wide range of cancer types [1–3]. In a series of studies using NORDCAN data for 1964–2003, we reported substantial improvements in cancer survival starting already in the 1960s [4–16]. In the most recent studies, we observed large improvements in survival for cancers of the colon, rectum, lung, kidney, breast, uterus, ovary and prostate, and melanoma of the skin [1]. The latest studies also documented that the previously observed survival gap in Denmark has diminished, and that cancer survival in Danish men and women is now in line with survival in the other Nordic countries.

The reasons for the improved cancer survival in the Nordic countries are likely multifactorial, including changes in healthcare policies from prevention to cancer control in general and resource allocation, as well as improved diagnostic measures and treatment options (Supplementary Table S1). Starting in the early 2000s, national cancer care guidelines and standardised patient pathways in cancer care were implemented in the Nordic countries to improve equal access to treatment and care across patient groups, which is imperative for efficient cancer control [17–19]. New diagnostic methods and cancer therapy, for example targeted treatments and immunotherapy, have been introduced during the recent 10–15 years [20, 21]. Increased screening activity and public awareness leading to early detection and optimisation of therapy are also likely to have influenced the trends. National screening programmes for breast and cervical cancer are well-established in the Nordic countries, and more recently national colorectal screening programmes have been introduced, starting in 2014 in Denmark.

Cancer survival trends using population-based cancer statistics provide, in combination with incidence and mortality trends, an important tool to evaluate effectiveness of cancer management in the population. Such trends also serve as much-needed benchmarks in comparisons between countries and regions [22]. A prerequisite is harmonised data definitions, data collection, and coding in well-defined health care settings. The improved diagnostic and therapeutic measures and the increasing cancer survival during the last two decades calls for continued monitoring. In addition, the favourable trends also raise the question on whether the survival improvements have been equal across all ages.

In the most recent update of NORDCAN (version 9.3), we have included age-specific survival, which extends on previous survival estimates in NORDCAN, and allows for careful evaluation of cancer survival trends by specific age groups [23]. These age-specific survival estimates are important since they can provide insight into underlying age patterns behind the increasing cancer survival in the Nordic population. Thereby, we can identify disadvantaged patient subgroups, for example by age, sex and cancer sites.

In the present study, we used the most recently updated NORDCAN data with diagnoses and follow-up through 2021. We aimed to assess whether the improvements in cancer survival during the last two decades have been similar across age groups. We also assessed which major cancer types have exhibited the most favourable survival trends.

## Materials and methods

Since 2002, the national cancer registries in Denmark, Faroe Islands, Finland, Greenland, Iceland, Norway and Sweden have published harmonised, high-quality, population-based cancer statistics via the NORDCAN database collaboration, which is a publicly available online resource (https://nordcan.iarc.fr/en/database) [23–25].

The quality of reported incidence data to the Nordic cancer registries has traditionally been high and almost complete, including 92%–97% morphologically verified diagnoses in 2017–2021 (men: ranging from 92.5% in Finland to 97.0% in Sweden; women: 92.2% in Finland to 97.4% in Sweden) (Supplementary Table S2). The proportions of cases reported by ‘death certificate only’ (DCO) were low, ranging from 0.0% (women in Iceland) to 1.8% (women in Finland), except for Sweden where no trace-back of death certificates has been performed due to legal reasons [24].

For the present study, we included 2,904,324 cancer cases (1,525,854 men, 1,378,470 women) diagnosed at ages 0–89 years between 2002 and 2021 for all countries except the Faroe Islands and Greenland (insufficient population sizes for analyses of age-specific survival trends). We also excluded in situ diagnoses. Multiple primaries were handled according to International Agency for Research on Cancer (IARC) multiple primary rules through the IARCcrg tool [26]. Cancer cases were followed for death until end of 2021.

We included all sites (excluding non-melanoma skin cancer) according to the NORDCAN entities based on the 10^th^ version of the International Classification of Diseases (ICD-10): C00–C97 (except C44, non-melanoma skin cancer) and D09.0–D09.1, D30.1–D30.9, D32–D33, D35.2–D35.4, D41.1–D41.9, D42–D43, D44.3–D44.5, D45–D47. Eight cancer sites were analysed in more detail: colorectal (C18–C20), pancreas (C25), lung (C33–C34), melanoma of skin (C43), and kidney (C64), in both sexes; breast (C50) and cervix uteri (C53), in women only; and prostate (C61) in men. The selected cancer sites represent sites for which there has been a development of diagnostic measures (such as screening) and new treatments (such as adjuvant treatments and immunotherapy) over the study period, as well as the four major cancer sites in the Nordic countries, that is breast, colorectal, lung, and prostate cancer.

### Statistical methods

Cancer incidence and mortality rates were defined as the number of events (new cases or deaths from cancer) divided by person-years based on annual mid-year populations Country-specific rates were estimated across 5-year age groups for men and women separately for 2017–2021.

We used relative survival (RS) as a measure of net survival, which was estimated at 5 year after diagnosis for patients aged 0–89 years at diagnosis using the Pohar-Perme estimator implemented in the Stata command stnet [27, 28]. Follow-up was defined as time from diagnosis until death or censoring due to first emigration after cancer or end of follow-up in December 31, 2021. For the last 5-year calendar period, where complete 5-year follow-up was not feasible, we applied the period approach. Death certificate only cases were excluded. The analysis was restricted to age groups with at least 30 patients alive at the start of follow-up, which meant that for Iceland, estimates of differences in 5-year RS could only be calculated for some cancer sites and age-groups due to low numbers. National life tables, stratified by sex, annual attained age and calendar year, were applied in the estimation of RS. We estimated RS 5 years after diagnosis, by country, cancer site, age group (0–49, 50–59, 60–69, 70–79, 80–89 years), and period of diagnosis (2002–2006, 2007–2011, 2012–2016, 2017–2021). Additionally, absolute differences in 5-year RS (expressed as percentage points, pp) were calculated comparing the earliest period (2002–2006) to the latest period (2017–2021), and presented by cancer site, age, sex, and country. Confidence intervals (CIs) for the pp differences were derived from the standard errors of the 5-year RS for each period and calculated as the sum of the two variances. These estimates are publicly available from the NORDCAN online database (https://nordcan.iarc.fr/en/, accessed on 10-11-2023). Statistical software Stata and R were used for the data analysis.

## Results

In total, 2,904,324 new cancer cases (1,525,854 men, 1,378,470 women) were diagnosed at ages 0–89 years in the Nordic countries during 2002–2021 (Table 1). The age distributions of cases were similar across the countries with the highest proportion of cases in age groups 60–69 and 70–79 years.

**Table 1:** Numbers of cancer cases (all sites of cancer, except non-melanoma of skin cancer) in 2002–2021 by country, sex, age and major sites.

Patient group	Denmark		Finland		Iceland		Norway		Sweden	
	*N*	%	*N*	%	*N*	%	*N*	%	*N*	%
**Total^a^**	715,176	100.0	586,677	100.0	29,351	100.0	563,514	100.0	1,009,606	100.0
**Sex**										
Women	349,678	48.9	283,828	48.4	14,309	48.8	262,142	46.5	468,513	46.4
Men	365,498	51.1	302,849	51.6	15,042	51.2	301,372	53.5	541,093	53.6
**Age**										
<50	77,463	10.8	56,260	9.6	3,962	13.5	64,942	11.5	98,238	9.7
50–59	107,156	15.0	86,859	14.8	4,600	15.7	82,936	14.7	135,184	13.4
60–69	200,076	28.0	159,988	27.3	7,478	25.5	148,435	26.3	279,226	27.7
70–79	211,021	29.5	168,901	28.8	7,933	27.0	154,859	27.5	306,749	30.4
80–89	119,460	16.7	114,669	19.5	5,378	18.3	112,342	19.9	190,209	18.8
**Major sites**										
Colorectal	89,084	12.5	59,362	10.1	3,100	10.6	78,921	14.0	121,967	12.1
Lung	90,022	12.6	51,712	8.8	3,359	11.4	57,422	10.2	77,538	7.7
Melanoma of skin	41,481	5.8	25,720	4.4	996	3.4	34,213	6.1	62,786	6.2
Kidney	16,336	2.3	17,750	3.0	1,096	3.7	15,099	2.7	21,940	2.2
Pancreas	19,245	2.7	22,759	3.9	759	2.6	14,869	2.6	24,049	2.4
Breast (women)	92,350	26.4^b^	89,100	31.4^b^	4,140	28.9^b^	62,410	23.8^b^	138,636	29.6^b^
Cervix uteri	7,322	2.1^b^	3,300	1.2^b^	326	2.3^b^	6,522	2.5^b^	9,976	2.1^b^
Prostate	82,866	22.7^c^	96,857	32.0^c^	4,321	28.7^c^	90,915	30.2^c^	197,764	36.5^c^

a All sites of cancer, except non-melanoma skin cancer.

b Percentage among women.

c Percentage among men.

### Incidence and mortality by age and country

The incidence rates during 2017–2021 increased sharply with age (Supplementary Figure S1). For ages below 50 years, the incidence was higher in women than in men, primarily due to cervical and breast cancer, while for ages above 50 years, men had a higher incidence. Among both men and women, cancer incidence was highest in Denmark and Norway across all age groups. Similarly, the mortality rates increased sharply by age with higher mortality in men than in women (Supplementary Figure S1). The selected cancer sites exhibited strong age-related increases in incidence (Supplementary Figure S2). For cancers of colorectum, lung, kidney, pancreas, and skin melanoma, women had a lower incidence than men at ages above 50 years.

### Trends in RS by age and country (all sites of cancer, except non-melanoma skin cancer)

**Figure 1:**
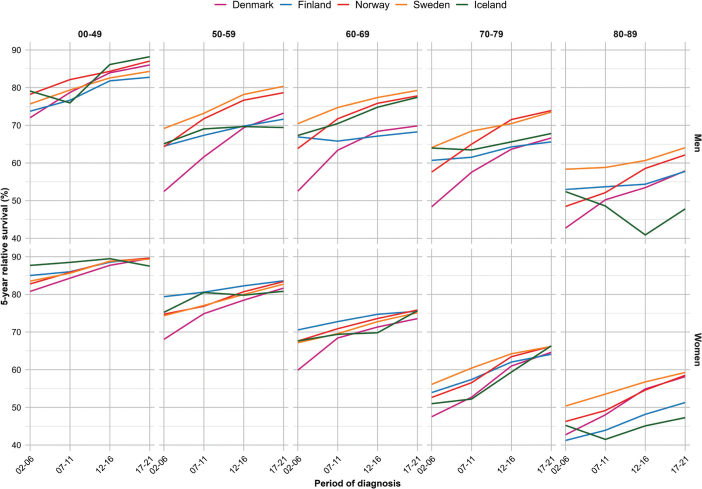
Five-year relative survival across year by country, age and sex for all sites of cancer (except non-melanoma of skin cancer).

**Figure 2:**
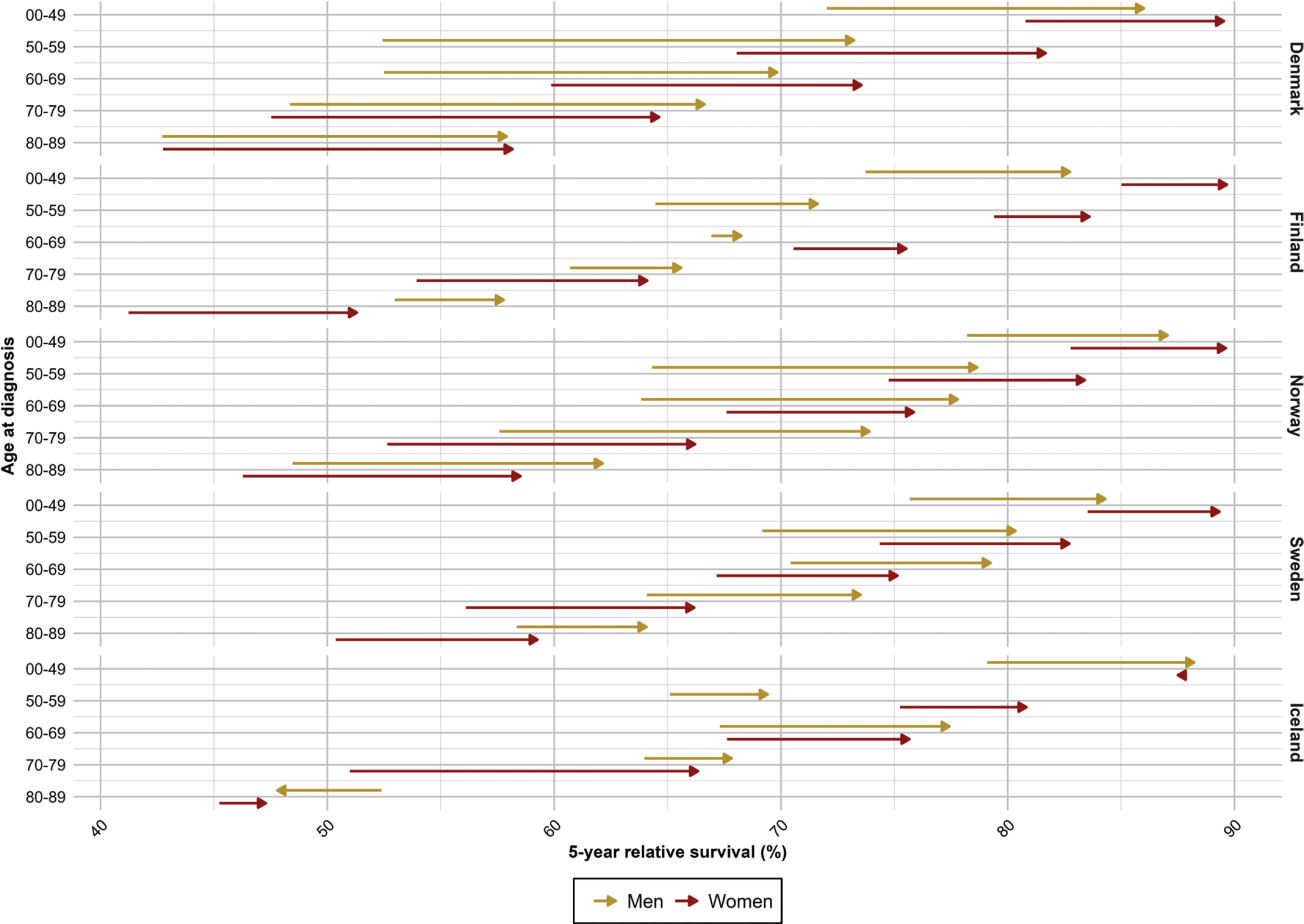
Survival improvements in five-year relative survival from 2002–2006 to 2017–2021 per country, age and sex for all sites of cancer (except non-melanoma of skin cancer).

For all sites combined, the 5-year RS increased over calendar period across all age groups and countries, except for patients aged 80–89 years in Iceland where random variation was larger due to fewer cases (Figure 1). We observed a strong age gradient with highest survival in younger patients, and lowest in older patients. In the latest period 2017–2021, patients below 50 years experienced a 5-year RS ranging from 82.7% (men, Finland) to 89.7% (women, Finland), while in the oldest patients (80–89 years), the corresponding RS ranged from 47.3% (women, Iceland) to 64.1% (men, Sweden) (Table 2). When comparing patients diagnosed in 2002–2006 to those diagnosed in 2017–2021, the RS improved across all ages and both sexes, with the most marked improvements in Denmark where the increases in survival ranged from 8.7 pp (women, <50 years) to 20.8 pp (men, 50–59 years) (Figure 2). The only exceptions from this trend were among Icelandic men aged 80–89 years and women aged <50 years who experienced no improvement in RS over the 20-year period.

**Table 2:** Age-specific five-year relative survival (RS) in latest period 2017–2021 and percentage point (PP) differences from 2002–2006 to 2017–2021. Men and women, Nordic countries.

Age at diagnosis	Denmark		Finland		Iceland		Norway		Sweden	
	RS (95% CI)	PP diff. (95% CI)	RS (95% CI)	PP diff. (95% CI)	RS (95% CI)	PP diff. (95% CI)	RS (95% CI)	PP diff. (95% CI)	RS (95% CI)	PP diff. (95% CI)
All sites,^a^ men										
<50	86.0 (85.2, 86.8)	14.0 (12.6, 15.4)	82.7 (81.7, 83.8)	9.0 (7.4, 10.7)	88.2 (85.0, 91.6)	9.1 (3.5, 14.7)	87.1 (86.2, 87.9)	8.8 (7.4, 10.2)	84.3 (83.6, 85.1)	8.6 (7.4, 9.9)
50–59	73.2 (72.4, 74.1)	20.8 (19.5, 22.1)	71.6 (70.6, 72.6)	7.2 (5.7, 8.6)	69.4 (65.3, 73.7)	4.3 (-2.3, 10.9)	78.7 (77.8, 79.5)	14.3 (12.9, 15.7)	80.3 (79.7, 81.0)	11.2 (10.2, 12.2)
60–69	69.8 (69.2, 70.5)	17.3 (16.3, 18.4)	68.3 (67.6, 69.0)	1.3 (0.2, 2.4)	77.4 (74.6, 80.4)	10.1 (5.3, 15.0)	77.8 (77.2, 78.4)	14.0 (12.9, 15.1)	79.2 (78.8, 79.7)	8.8 (8.1, 9.6)
70–79	66.6 (65.9, 67.4)	18.3 (17.1, 19.4)	65.6 (64.8, 66.4)	4.9 (3.7, 6.1)	67.8 (64.1, 71.8)	3.8 (-1.7, 9.4)	73.9 (73.1, 74.7)	16.3 (15.0, 17.6)	73.5 (73.0, 74.1)	9.4 (8.5, 10.3)
80–89	57.9 (56.2, 59.7)	15.2 (12.6, 17.7)	57.8 (56.1, 59.5)	4.8 (2.1, 7.5)	47.8 (40.8, 56.0)	-4.6 (-15.8, 6.7)	62.1 (60.3, 64.0)	13.7 (11.1, 16.3)	64.1 (62.7, 65.4)	5.7 (3.8, 7.6)
All sites,^a^ women										
<50	89.5 (89.0, 90.1)	8.7 (7.8, 9.7)	89.7 (89.0, 90.3)	4.6 (3.6, 5.7)	87.5 (84.9, 90.2)	-0.2 (-3.9, 3.6)	89.6 (89.0, 90.2)	6.8 (5.8, 7.9)	89.3 (88.9, 89.8)	5.8 (5.0, 6.6)
50–59	81.7 (81.0, 82.4)	13.6 (12.5, 14.7)	83.6 (82.9, 84.3)	4.2 (3.1, 5.3)	80.8 (77.7, 84.1)	5.6 (0.5, 10.6)	83.4 (82.7, 84.2)	8.6 (7.5, 9.8)	82.7 (82.1, 83.3)	8.4 (7.5, 9.3)
60–69	73.6 (72.9, 74.2)	13.7 (12.6, 14.7)	75.5 (74.8, 76.2)	5.0 (3.8, 6.1)	75.7 (72.6, 78.9)	8.0 (2.8, 13.3)	75.9 (75.1, 76.6)	8.3 (7.0, 9.5)	75.1 (74.6, 75.7)	8.0 (7.1, 8.9)
70–79	64.6 (63.9, 65.4)	17.1 (15.9, 18.3)	64.1 (63.3, 65.0)	10.2 (8.8, 11.5)	66.3 (62.2, 70.8)	15.4 (8.9, 21.8)	66.2 (65.3, 67.1)	13.6 (12.1, 15)	66.2 (65.6, 66.8)	10.1 (9.1, 11.1)
80–89	58.2 (56.6, 59.7)	15.4 (13.2, 17.6)	51.3 (49.9, 52.8)	10.1 (7.9, 12.2)	47.3 (41.1, 54.4)	2.0 (-8.2, 12.3)	58.5 (56.9, 60.2)	12.2 (9.9, 14.5)	59.3 (58.1, 60.5)	8.9 (7.2, 10.6)
Colorectal, men										
<50	73.7 (70.0, 77.6)	12.7 (6.8, 18.7)	73.1 (69.1, 77.4)	7.4 (1.1, 13.7)	77.8 (64.9, 93.2)	N/A^b^	76.7 (73.5, 80.1)	9.7 (4.1, 15.3)	72.8 (70.0, 75.8)	7.6 (2.7, 12.4)
50–59	75.1 (72.8, 77.5)	16.1 (12.5, 19.7)	68.9 (65.9, 72.1)	10.3 (5.7, 14.9)	68.9 (59.1, 80.3)	-7.3 (-24.2, 9.5)	70.3 (67.7, 72.9)	8.8 (4.8, 12.8)	70.9 (68.7, 73.3)	7.9 (4.5, 11.3)
60–69	74.3 (72.7, 75.9)	17.0 (14.3, 19.7)	68.1 (66.1, 70.3)	1.8 (-1.6, 5.2)	73.7 (65.1, 83.4)	4.2 (-10.1, 18.4)	72.6 (70.8, 74.5)	10.4 (7.4, 13.4)	69.8 (68.2, 71.3)	8.0 (5.6, 10.4)
70–79	72.9 (71.2, 74.6)	20.2 (17.4, 23.1)	65.1 (62.9, 67.4)	7.6 (4.0, 11.2)	69.6 (59.9, 80.8)	6.7 (-8.7, 22.1)	70.0 (68.1, 72.0)	11.8 (8.7, 14.9)	70.0 (68.6, 71.5)	12.1 (9.7, 14.5)
80–89	68.4 (64.4, 72.6)	18.9 (12.8, 25.0)	62.9 (58.7, 67.3)	13.2 (5.8, 20.6)	61.8 (44.5, 85.9)	-1.5 (-31.3, 28.3)	67.8 (64.0, 71.8)	13.5 (7.7, 19.2)	65.0 (62.1, 68.2)	9.5 (5.0, 14.0)
Colorectal, women										
<50	77.8 (74.3, 81.4)	13.1 (7.2, 18.9)	81.9 (78.4, 85.5)	5.8 (0.5, 11.1)	69.1 (56.3, 84.8)	N/A^b^	77.0 (73.9, 80.2)	10.2 (4.8, 15.6)	77.3 (74.6, 80.2)	10.8 (6.0, 15.7)
50–59	79.0 (76.7, 81.4)	17.8 (14.1, 21.6)	74.2 (71.1, 77.4)	5.1 (0.5, 9.7)	82.9 (72.5, 94.7)	12.9 (-6.4, 32.1)	76.4 (73.8, 79.2)	9.1 (5.0, 13.2)	73.9 (71.6, 76.4)	9.2 (5.6, 12.7)
60–69	76.4 (74.6, 78.2)	15.0 (12.1, 18.0)	72.4 (70.1, 74.6)	5.6 (2.0, 9.2)	74.3 (65.8, 83.9)	-5.5 (-20.7, 9.7)	73.1 (71.2, 75.2)	6.2 (3.0, 9.4)	71.4 (69.8, 73.1)	5.7 (3.1, 8.3)
70–79	71.5 (69.7, 73.3)	15.7 (12.8, 18.6)	70.3 (68.1, 72.5)	8.6 (5.1, 12.2)	70.5 (60.2, 82.5)	9.8 (-6.6, 26.2)	70.5 (68.6, 72.4)	8.8 (5.7, 11.8)	69.5 (68.0, 71.1)	7.0 (4.6, 9.4)
80–89	62.6 (59.4, 66.0)	11.3 (6.6, 16.0)	66.2 (62.7, 70.0)	11.6 (6.0, 17.1)	45.2 (33.5, 60.9)	-12.9 (-36.3, 10.5)	69.7 (66.5, 72.9)	11.8 (7.3, 16.3)	65.7 (63.2, 68.3)	3.9 (0.2, 7.7)
Lung, men										
<50	40.7 (34.4, 48.3)	26.9 (19.1, 34.6)	36.8 (29.4, 46.1)	15.8 (5.8, 25.8)	N/A^c^	N/A^c^	39.8 (32.9, 48.3)	13.7 (4.2, 23.2)	39.3 (32.9, 47.0)	16.4 (7.8, 25.1)
50–59	26.2 (23.6, 29.1)	15.2 (12.0, 18.4)	17.3 (14.7, 20.4)	2.3 (-1.2, 5.9)	38.4 (26.6, 55.4)	22.5 (5.2, 39.8)	30.3 (27.0, 34.2)	13.8 (9.5, 18.1)	27.8 (24.5, 31.5)	13.4 (9.4, 17.5)
60–69	24.0 (22.5, 25.6)	14.1 (12.2, 16.0)	16.7 (15.2, 18.2)	5.7 (3.7, 7.8)	33.2 (25.3, 43.7)	24.3 (13.1, 35.4)	26.5 (24.6, 28.4)	16.1 (13.7, 18.4)	25.9 (24.1, 27.8)	13.3 (11.0, 15.5)
70–79	23.6 (22.1, 25.1)	15.2 (13.4, 17.0)	15 (13.6, 16.5)	8.2 (6.4, 10.0)	19.4 (13.0, 28.8)	4.4 (-5.8, 14.5)	25.5 (23.7, 27.4)	15.6 (13.3, 17.9)	22.8 (21.4, 24.4)	11.4 (9.4, 13.3)
80–89	16.9 (14.4, 19.9)	13.5 (10.4, 16.6)	5.1 (3.8, 6.9)	3.8 (2.0, 5.6)	13.3 (4.7, 37.2)	3.3 (-14.6, 21.1)	19.5 (16.5, 23.0)	16.1 (12.6, 19.7)	13.5 (11.3, 16.1)	7.5 (4.5, 10.5)
Lung, women										
<50	47.3 (41.5, 53.9)	27.7 (20.6, 34.9)	44.2 (35.3, 55.3)	15.8 (3.6, 28.0)	N/A^c^	N/A^c^	47.4 (41.0, 54.7)	20.5 (11.6, 29.4)	49.1 (43.1, 56.0)	16.1 (8.0, 24.3)
50–59	36.3 (33.8, 39.0)	20.6 (17.4, 23.8)	31.0 (26.9, 35.7)	11.5 (6.0, 17.1)	27.4 (17.9, 42.0)	13.1 (-1.5, 27.6)	38.0 (34.6, 41.7)	21.1 (16.8, 25.5)	38.0 (34.9, 41.4)	19.9 (16.1, 23.8)
60–69	33.3 (31.7, 35.0)	20.9 (18.8, 23.0)	25.5 (23.3, 27.8)	8.2 (4.5, 11.8)	41.8 (33.9, 51.6)	26.3 (14.7, 37.9)	36.3 (34.3, 38.6)	18.5 (15.4, 21.6)	33.9 (32.2, 35.6)	16.4 (14.0, 18.8)
70–79	28.1 (26.6, 29.7)	19.3 (17.4, 21.2)	22.9 (20.8, 25.2)	12.1 (9.0, 15.3)	33.7 (25.7, 44.0)	22.5 (11.4, 33.7)	33.1 (31.2, 35.2)	21.0 (18.3, 23.7)	28.8 (27.4, 30.3)	14.0 (11.8, 16.2)
80–89	21.1 (18.6, 24.0)	16.6 (13.4, 19.8)	11.7 (9.2, 14.8)	7.1 (3.6, 10.6)	17.5 (9.4, 32.7)	12.2 (-0.9, 25.3)	18.7 (15.7, 22.2)	11.1 (6.9, 15.3)	20.8 (18.2, 23.6)	12.9 (9.5, 16.4)
Melanoma of skin, men										
<50	97.4 (96.4, 98.5)	7.8 (5.3, 10.4)	93.4 (91.3, 95.5)	7.5 (3.4, 11.6)	N/A^c^	N/A^c^	95.8 (94.3, 97.4)	9.4 (6.0, 12.7)	96.7 (95.7, 97.7)	6.6 (4.4, 8.8)
50–59	97.0 (95.5, 98.5)	9.6 (6.1, 13.0)	92.5 (90.2, 94.9)	9.6 (4.9, 14.2)	N/A^c^	N/A^c^	93.3 (91.4, 95.2)	12.7 (8.7, 16.8)	94.2 (92.9, 95.6)	9.9 (7.0, 12.7)
60–69	94.9 (92.9, 96.9)	13.5 (9.5, 17.6)	90.2 (87.8, 92.7)	9.4 (4.5, 14.3)	N/A^c^	N/A^c^	89.5 (87.4, 91.7)	13.4 (8.8, 18.0)	92.5 (91.1, 94.0)	8.4 (5.4, 11.3)
70–79	92.1 (89.5, 94.7)	13.5 (7.0, 20.0)	91.3 (88.2, 94.4)	17.7 (11.0, 24.4)	N/A^c^	N/A^c^	90.1 (87.5, 92.8)	18.2 (12.3, 24.0)	92.7 (90.8, 94.5)	12.7 (8.7, 16.8)
80–89	96.2 (88.2, 105.0)	30.1 (14.3, 45.9)	96.8 (89.3, 104.9)	16.8 (1.1, 32.6)	N/A^c^	N/A^c^	81.0 (74.7, 87.9)	15.4 (3.1, 27.7)	88.8 (83.9, 94.0)	11.0 (2.0, 19.9)
Melanoma of skin, women										
<50	98.4 (97.8, 99.0)	2.7 (1.4, 4.0)	97.5 (96.4, 98.5)	3.4 (1.0, 5.8)	93.8 (86.8, 101.3)	-5.4 (-13, 2.1)	97.7 (96.8, 98.6)	4.6 (2.7, 6.5)	97.3 (96.6, 98.0)	2.7 (1.3, 4.1)
50–59	97.6 (96.4, 98.7)	3.7 (1.2, 6.1)	96.5 (94.8, 98.2)	6.6 (2.7, 10.4)	90.3 (79.0, 103.2)	N/A^b^	96.6 (95.2, 98.0)	7.2 (4.1, 10.4)	97.0 (95.9, 98.0)	4.9 (2.7, 7.0)
60–69	97.9 (96.4, 99.5)	7.8 (4.3, 11.3)	94.1 (92.0, 96.2)	3.2 (-1.0, 7.3)	N/A^c^	N/A^c^	96.4 (94.8, 98.0)	7.6 (3.9, 11.3)	96.5 (95.2, 97.7)	8.1 (5.3, 10.8)
70–79	96.4 (94.1, 98.9)	11.0 (5.3, 16.6)	90.9 (87.9, 94.1)	10.6 (4.4, 16.7)	N/A^c^	N/A^c^	95.2 (92.7, 97.7)	12.7 (7.4, 18.1)	94.6 (92.8, 96.4)	9.6 (5.8, 13.5)
80–89	95.2 (88.5, 102.4)	14.5 (2.7, 26.2)	89.3 (82.3, 97.0)	16.7 (3.9, 29.6)	N/A^c^	N/A^c^	88.8 (82.8, 95.3)	6.0 (-5.1, 17.2)	86.8 (82.3, 91.6)	-2.5 (-10.6, 5.7)
Kidney, men										
<50	86.3 (82.4, 90.3)	18.3 (10.5, 26.1)	88.6 (84.5, 92.9)	16.0 (8.4, 23.6)	N/A^c^	N/A^c^	86.8 (83.2, 90.6)	12.5 (5.3, 19.8)	86.8 (83.6, 90.2)	9.8 (3.6, 16.0)
50–59	77.9 (74.4, 81.5)	26.0 (19.8, 32.3)	73.6 (69.4, 78.0)	9.4 (2.9, 15.9)	83.6 (72.4, 96.6)	N/A^b^	81.8 (78.5, 85.2)	16.4 (10.2, 22.6)	83.1 (80.1, 86.2)	21.2 (15.7, 26.7)
60–69	76.8 (73.7, 80.0)	26.7 (21.1, 32.4)	73.2 (69.8, 76.7)	11.6 (5.7, 17.5)	81.8 (70.8, 94.6)	N/A^b^	82.5 (79.6, 85.6)	22.4 (16.4, 28.3)	81.3 (78.7, 83.9)	20.1 (15.5, 24.8)
70–79	75.0 (71.3, 78.9)	30.6 (23.5, 37.6)	66.4 (62.4, 70.7)	5.4 (-1.5, 12.4)	81.4 (65.8, 100.7)	7.1 (-17.3, 31.4)	75.4 (71.5, 79.4)	19.1 (12.1, 26.1)	78.0 (75.0, 81.1)	20.5 (14.9, 26.0)
80–89	75.1 (64.0, 88.0)	44.0 (27.1, 61.0)	68.8 (59.2, 79.9)	28.8 (13.3, 44.2)	N/A^c^	N/A^c^	62.1 (52.2, 73.9)	12.7 (-2.7, 28.2)	77.2 (68.8, 86.7)	21.6 (7.7, 35.5)
Kidney, women										
<50	94.3 (90.4, 98.3)	26.0 (15.0, 37.0)	94.0 (89.7, 98.4)	7.1 (-0.2, 14.5)	N/A^c^	N/A^c^	88.7 (84.1, 93.5)	1.9 (-6.1, 9.9)	93.6 (90.5, 96.9)	15.5 (8.2, 22.8)
50–59	84.5 (80.1, 89.2)	27.1 (18.2, 35.9)	85.6 (81.1, 90.4)	11.4 (4.2, 18.6)	N/A^c^	N/A^c^	88.4 (83.7, 93.3)	15.7 (6.4, 25.0)	87.9 (84.2, 91.8)	18.3 (11.4, 25.1)
60–69	79.8 (75.7, 84.2)	33.4 (25.7, 41.2)	74.5 (70.5, 78.8)	8.4 (1.5, 15.4)	90.3 (79.8, 102.1)	N/A^b^	81.0 (76.7, 85.5)	12.2 (4.3, 20.1)	80.9 (77.3, 84.6)	19.3 (13.4, 25.2)
70–79	70.9 (66.1, 76.1)	26.2 (17.9, 34.5)	72.0 (67.6, 76.6)	12.9 (6.0, 19.7)	66.5 (47.1, 93.9)	N/A^b^	75.6 (70.5, 81.0)	19.0 (10.8, 27.2)	74.5 (70.8, 78.5)	17.9 (11.7, 24.1)
80–89	62.6 (52.7, 74.4)	36.7 (21.9, 51.5)	56.0 (48.7, 64.3)	11.9 (0.6, 23.3)	N/A^c^	N/A^c^	69.9 (58.6, 83.4)	30.0 (14.3, 45.7)	69.3 (61.3, 78.3)	15.3 (2.4, 28.2)
Pancreas, men										
<50	29.4 (21.6, 40.1)	21.8 (11.3, 32.2)	41.1 (31.7, 53.3)	27.2 (14.7, 39.7)	N/A^c^	N/A^c^	34.2 (24.7, 47.4)	19.3 (5.3, 33.3)	37.8 (30.6, 46.7)	13.9 (2.1, 25.7)
50–59	16.2 (12.4, 21.3)	9.7 (4.5, 14.8)	10.8 (7.9, 14.8)	4.7 (0.4, 8.9)	N/A^c^	N/A^c^	24.8 (19.6, 31.5)	14.9 (7.8, 22.0)	15.4 (12.1, 19.6)	8.7 (4.2, 13.3)
60–69	10.7 (8.5, 13.4)	7.5 (4.6, 10.3)	9.1 (7.3, 11.3)	4.9 (2.2, 7.5)	N/A^c^	N/A^c^	13.9 (11.0, 17.6)	9.4 (5.5, 13.3)	11.4 (9.5, 13.6)	6.9 (4.3, 9.6)
70–79	8.3 (6.5, 10.6)	5.9 (3.4, 8.3)	5.9 (4.5, 7.8)	3.2 (1.1, 5.4)	N/A^c^	N/A^c^	12.3 (9.8, 15.4)	9.5 (6.2, 12.8)	10.5 (8.8, 12.4)	7.7 (5.4, 9.9)
80–89	3.9 (2.0, 7.6)	1.8 (-1.9, 5.4)	1.3 (0.5, 3.5)	-0.7 (-3.3, 1.9)	N/A^c^	N/A^c^	10.1 (6.1, 16.6)	6.2 (0.2, 12.2)	8.7 (6.0, 12.7)	8.0 (4.5, 11.6)
Pancreas, women										
<50	35.4 (26.1, 48.1)	9.9 (-4.6, 24.4)	48.8 (39.4, 60.5)	25.1 (10.7, 39.5)	N/A^c^	N/A^c^	49.3 (38.8, 62.7)	36.1 (22, 50.2)	44.6 (37.0, 53.7)	29.2 (17.9, 40.5)
50–59	22.0 (17.2, 28.2)	16.6 (10.5, 22.6)	14.2 (10.4, 19.6)	5.5 (-0.2, 11.3)	N/A^c^	N/A^c^	24.6 (18.6, 32.6)	16.2 (8.1, 24.3)	21.0 (17.1, 25.7)	13.1 (7.9, 18.3)
60–69	11.1 (8.6, 14.3)	7.5 (4.3, 10.8)	10.4 (8.3, 12.9)	5.6 (2.6, 8.6)	N/A^c^	N/A^c^	15.3 (11.9, 19.8)	11.6 (7.1, 16.1)	15.2 (12.8, 18.0)	9.5 (6.3, 12.7)
70–79	8.6 (6.7, 11.0)	6.8 (4.4, 9.2)	6.4 (5.0, 8.1)	3.6 (1.6, 5.6)	N/A^c^	N/A^c^	10.7 (8.2, 14.0)	7.3 (3.9, 10.7)	9.8 (8.2, 11.8)	6.6 (4.4, 8.9)
80–89	3.5 (1.8, 6.6)	2.7 (0.2, 5.2)	1.4 (0.7, 2.7)	-0.1 (-1.7, 1.5)	N/A^c^	N/A^c^	3.2 (1.7, 6.2)	2.3 (-0.1, 4.7)	4.5 (2.9, 7.1)	3.0 (0.4, 5.5)
Prostate, men										
<50	92.8 (88.8, 96.9)	23.1 (11.4, 34.9)	91.5 (87.5, 95.7)	-1.9 (-7.4, 3.6)	N/A^c^	N/A^c^	97.4 (95.3, 99.5)	10.2 (3.8, 16.5)	96.9 (95.3, 98.6)	7.7 (3.6, 11.8)
50–59	94.0 (92.8, 95.3)	14.3 (11.6, 17.1)	96.7 (95.7, 97.7)	1.5 (0.0, 2.9)	93.4 (87.7, 99.5)	-0.5 (-9.1, 8.1)	97.0 (96.1, 97.8)	5.8 (4.1, 7.5)	97.4 (96.8, 98.0)	2.8 (1.8, 3.8)
60–69	93.9 (93.0, 94.7)	11.7 (10.0, 13.3)	95.5 (94.8, 96.3)	1.1 (-0.1, 2.2)	97.4 (94.6, 100.4)	5.4 (0.2, 10.7)	97.9 (97.3, 98.5)	6.3 (5.1, 7.5)	97.9 (97.4, 98.3)	4.2 (3.5, 4.9)
70–79	90.0 (88.9, 91.2)	15.2 (13.1, 17.3)	95.0 (93.9, 96.0)	3.1 (1.4, 4.7)	93.5 (88.6, 98.8)	7.3 (-0.3, 14.8)	97.6 (96.6, 98.5)	14.1 (12.2, 15.9)	95.5 (94.8, 96.2)	7.9 (6.7, 9.1)
80–89	77.3 (73.7, 81.1)	12.4 (7.2, 17.7)	88.0 (85.1, 91.0)	4.0 (-0.6, 8.6)	71.3 (56.8, 89.5)	2.0 (-19, 23.1)	79.1 (75.8, 82.5)	9.8 (5.1, 14.5)	84.8 (82.5, 87.1)	5.9 (2.7, 9.1)
Breast, women										
<50	92.0 (91.1, 92.9)	3.8 (2.4, 5.2)	92.3 (91.3, 93.2)	1.4 (0.0, 2.7)	87.3 (82.9, 91.9)	-2.7 (-9.0, 3.6)	93.5 (92.6, 94.4)	5.8 (4.2, 7.3)	94.0 (93.4, 94.6)	2.8 (1.8, 3.8)
50–59	94.5 (93.8, 95.3)	7.6 (6.3, 8.9)	94.3 (93.6, 95.0)	0.4 (-0.6, 1.4)	92.7 (89.2, 96.2)	-1.1 (-6.3, 4.0)	95.1 (94.3, 95.8)	3.1 (1.8, 4.3)	94.8 (94.2, 95.4)	2.7 (1.8, 3.6)
60–69	94.7 (93.9, 95.4)	8.6 (7.2, 10.0)	95.3 (94.6, 95.9)	3.3 (2.1, 4.4)	95.3 (92.0, 98.7)	7.4 (1.2, 13.7)	95.2 (94.4, 96.1)	3.2 (1.7, 4.6)	95.4 (94.8, 96.0)	3.7 (2.8, 4.7)
70–79	87.1 (85.7, 88.5)	8.1 (5.7, 10.5)	89.1 (87.9, 90.4)	7.5 (5.2, 9.8)	93.2 (87.3, 99.6)	11.1 (-0.2, 22.5)	88.2 (86.4, 89.9)	7.2 (4.2, 10.2)	93.1 (92.2, 94.0)	8.6 (6.9, 10.3)
80–89	82.4 (79.2, 85.7)	7.9 (2.9, 12.9)	80.9 (77.9, 84.0)	5.2 (0.1, 10.3)	83.3 (68.7, 101.1)	6.5 (-18.8, 31.9)	80.8 (76.8, 84.9)	4.5 (-1.3, 10.2)	82.7 (80.2, 85.3)	8.6 (5.0, 12.3)
Cervix, women										
<50	90.2 (88.3, 92.2)	3.4 (0.5, 6.3)	87.9 (84.8, 91.1)	0.5 (-4.2, 5.3)	90.2 (81.2, 100.2)	N/A^b^	90.3 (88.5, 92.1)	2.2 (-0.8, 5.1)	91.6 (90.2, 93.0)	4.0 (1.6, 6.5)
50–59	78.5 (73.4, 83.9)	8.6 (1.0, 16.2)	68.1 (60.7, 76.3)	2.5 (-9.1, 14.2)	N/A^c^	N/A^c^	80.7 (75.8, 85.9)	7.6 (0.0, 15.1)	77.6 (73.4, 82.1)	3.0 (-3.5, 9.6)
60–69	76.0 (70.1, 82.4)	26.4 (17.3, 35.6)	60.6 (52.2, 70.3)	4.9 (-9.7, 19.6)	N/A^c^	N/A^c^	77.6 (71.9, 83.8)	10.7 (1.2, 20.2)	72.4 (67.1, 78.1)	11.3 (3.1, 19.4)
70–79	62.3 (54.9, 70.6)	23.4 (12.7, 34.2)	54.8 (44.2, 67.9)	11.0 (-4.5, 26.5)	N/A^c^	N/A^c^	57.0 (48.1, 67.5)	3.7 (-9.9, 17.3)	54.9 (48.9, 61.7)	11.3 (2.2, 20.4)
80–89	35.7 (23.9, 53.1)	12.8 (-4.7, 30.3)	43.0 (27.1, 68.4)	10.3 (-14, 34.7)	N/A^c^	N/A^c^	34.5 (22.0, 54.1)	-10.9 (-32.0, 10.3)	32.4 (24.2, 43.5)	2.2 (-10.6, 14.9)

pp: percentage points

a All sites of cancer, except non-melanoma skin cancer.

b N/A as Not Available, meaning that survival estimates could be computed for the latest period, but not for the first. That is the difference cannot be calculated.

c N/A as Not Available, meaning that survival estimates could not be computed, as did not meet requirement of 30 patients alive at start.

### Site-specific trends in RS by age and country

**Figure 3:**
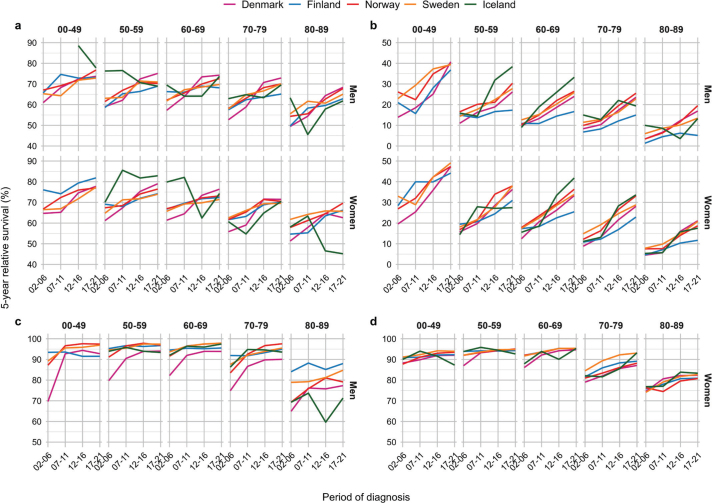
Five-year relative survival across year by country, age and sex by major cancer sites. Panel (A) colorectal, (B) lung, (C) prostate, and (D) breast.

**Figure 4:**
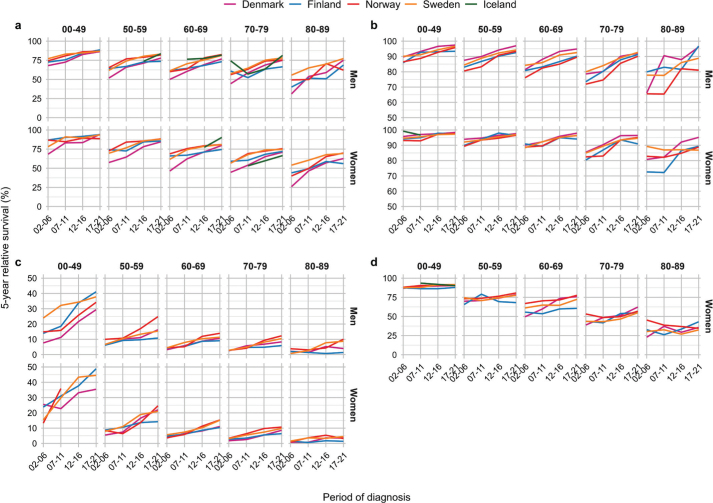
Five-year relative survival across year by country, age and sex by major cancer sites. Panel (A) kidney, (B) melanoma of skin, (C) pancreas and (D) cervix uteri.

**Figure 5:**
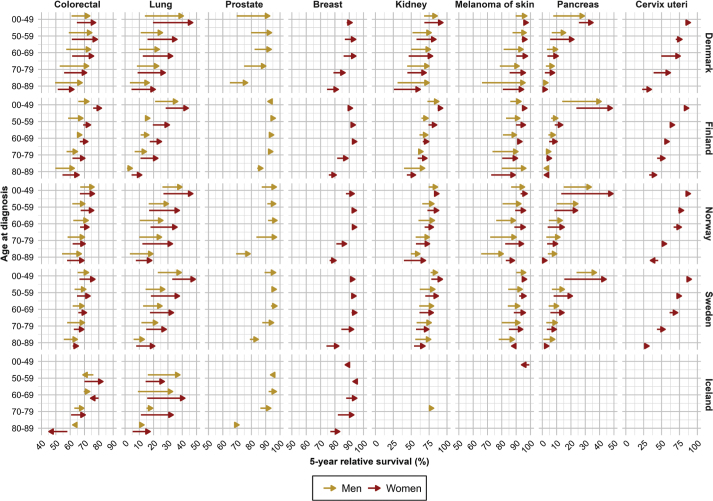
Survival improvements in five-year relative survival from 2002–2006 to 2017–2021 per country, age and sex by major cancer sites.

Five-year RS increased consistently from 2002 until 2021 across cancer sites and age groups in all countries, except for Iceland where the trends were less clear (Figures 3 and 4). Absolute percentage point differences in 5-year RS between diagnostic periods 2002–2006 and 2017–2021 ranged from 2 to 20 pp (colorectum), -1 to 36 pp (pancreas), 2 to 28 pp (lung), 0 to 9 pp (breast), -11 to 26 pp (cervix uteri), 2 to 44 pp (kidney), -2 to 23 pp (prostate) and -3 to 30 pp (skin melanoma) across age groups (Table 2, Figure 5). The survival trends are also displayed by age groups, across countries and cancer site, to highlight age differences (Supplementary Figures S3 and S4).

### Colorectal cancer

For colorectal cancer, the 5-year RS increased over time across all ages in all countries, except in Iceland, (Figure 3A). Danish colorectal cancer patients aged 50–59 years experienced the highest 5-year RS, while the highest RS in the other countries was observed among patients aged <50 years (Table 2). In the latest period, patients aged 50–69 years exhibited a 5-year RS ranging from 68% to 83% in all countries, while the oldest patients experienced lower survival.

From diagnostic period 2002–2006 to 2017–2021, the 5-year RS improved in all ages and both sexes, with the most substantial improvements in Denmark (Figure 5). Improvements also occurred in Norway and Sweden, and to some extent in Finland. In Iceland, the trends were less consistent.

### Lung cancer

For lung cancer, the 5-year RS increased sharply during the entire study period, in particular in younger patients and among women (Figure 3B). Among patients above 70 years, the RS increased less over time. In the latest period, patients below 50 years experienced 5-year RS ranging from 37% to 49% (Table 2). Among the eldest lung cancer patients (80–89 years), the RS ranged from 5% to 21% in the latest period. Patients in Finland experienced lowest RS in the most recent period among both sexes and all ages, except among women aged 50–59 years.

Compared to 2002–2006, Danish lung cancer patients diagnosed in 2017–2021 experienced substantial improvements in cancer survival among both sexes, and substantial improvements were also observed in Norway, Sweden and Iceland among both sexes and across all ages (Figure 5). In Finland, the survival improvements were smaller, especially among men.

### Prostate cancer

Five-year RS of prostate cancer increased from 2002–2006 to 2007–2011, especially in Denmark, reaching a plateau ranging from 87% to 98% in all age groups below 80 years (Figure 3C). Across all countries, the oldest men aged 80–89 years experienced the lowest RS. Yet, in Finland the oldest men had a RS of 88% in the latest period, while the corresponding RS estimates in Iceland and Denmark, that is the countries presenting the lowest survival, were 71% and 77%, respectively (Table 2).

Compared to 2002–2006, the 5-year RS improved substantially in Danish men diagnosed with prostate cancer in 2017–2021 (Figure 5). The improvement in prostate cancer survival was more modest in the other Nordic countries, reflecting the markedly lower survival in Danish prostate cancer patients initially in the study period compared to patients in the other Nordic countries. In all countries, the oldest men aged 80–89 years experienced only small improvements in cancer survival during the study period and remained at lower 5-year RS than patients in other ages.

### Breast cancer

For breast cancer in women, the 5-year RS was highest among patients aged 50–69 years, ranging from 93% to 95% in the latest period. The RS was lower among patients below 50 years, yet with lowest RS was in older women aged 80–89 years (Figure 3D). However, RS improved over time, in particularly in the oldest age groups, exhibiting a consistent pattern across countries. The increase in RS was, however, was less pronounced in the latest period across most ages.

In comparison to 2002–2006, the largest survival improvements among breast cancer patients diagnosed in 2017–2021 were observed in ages 70–79 and 80–89 years (Figure 5). In Denmark, improvements in cancer survival were also observed in women aged 50–59 and 60–69 years. In Iceland, cancer survival declined somewhat in women below 60 years, although from high levels.

### Kidney cancer

The 5-year RS for kidney cancer was highest among patients below 50 years, ranging from 86% to 94% in the latest period (Table 2). The RS generally increased during the study period, although the increase was less pronounced in the most recent period (Figure 4A). In 2002–2006, RS was consistently lowest in Denmark for both sexes and across all age groups.

When comparing kidney cancer patients diagnosed in 2002–2006 and 2017–2021, we observed substantial improvements in RS in Denmark, and also among kidney cancer patients in Finland, Norway and Sweden (Figure 5). The improvements were consistent across age groups, and in particular among the oldest patients (80–89 years).

### Melanoma of skin

For melanoma of the skin, the 5-year RS was higher among women than men (Figure 4B). During the study period, men experienced a larger increase in RS than women, reducing the sex difference in RS in the latest period. The RS for melanoma of skin was highest in the youngest patients below 50 years, ranging from 93% to 98% in the latest period.

When comparing patients diagnosed in 2002–2006 and 2017–2021, we observed substantial improvements in RS of skin melanoma in Denmark, particularly among the oldest patients and among men (Figure 5). Similarly, RS for skin melanoma improved in Finland and Norway, in particular among men. However, Norwegian men aged 80–89 years at diagnosis had substantially lower RS compared to the other countries in the latest period.

### Pancreatic cancer

For pancreatic cancer, the 5-year RS increased sharply during the study period among younger patients below 50 years, while the increase in RS was less prominent in older patients (Figure 4C). Among younger patients, the RS ranged from 29% to 49% in the latest period, with lowest RS in Denmark. Among patients aged 50–59 years, the RS ranged from 11% to 25%, while patients above 60 years experienced some improvements, although the RS was consistently below 20% (Table 2).

When comparing patients diagnosed in 2002–2006 to those diagnosed in 2017–2021, the largest improvements in cancer survival were observed in younger patients below 50 years and in women (Figure 5). Some improvements also occurred in other age groups, notably among patients aged 50–59 years in Denmark, Norway and Sweden.

### Cancer of cervix uteri

Five-year RS of cervix cancer was highest among women below 50 years, with consistently declining RS across age (Figure 4D). In the latest period, the RS among young women ranged from 88% to 92%, while the oldest women (80–89 years) experienced a RS ranging from 32% to 43%, highest in Finland (Table 2). However, women aged 50–69 years in Finland experienced somewhat lower RS compared to the other countries.

The comparison of 2002–2006 and 2017–2021 exhibited the largest increases in RS for cervical cancer in Denmark, in particular in women above 60 years (Figure 5). Only minor improvements in cancer survival were seen in the other Nordic countries.

## Discussion

In this large population-based study covering 27 million residents in the Nordic countries, we found large consistent improvements in cancer survival across age groups over the last 20 years. Five-year RS increased over time for most cancer sites, with positive trends present across cancers with high survival, for example breast and prostate cancer, as well as cancers with poorer survival, for example lung and pancreatic cancer. Despite these general improvements over age, older patients experienced lower survival than younger patients. For example, older patients with breast or prostate cancer exhibited substantially lower survival than younger patients throughout the study period in all countries. For some cancer sites, for example cervix, kidney and lung, an age gradient was more prominent with lower survival also among the middle-aged. Overall, our results thus indicate that the improvements in cancer survival have occurred in most age groups, although not equally for all cancer types. We found no substantial variation in age patterns of survival between women and men, although women tended to have a higher survival for most cancer sites.

During the study period, national initiatives have been implemented in the Nordic countries to standardise cancer therapy, for example clinical care guidelines with uniform therapeutic recommendations (Supplementary Table S1). In addition, standardised patient pathways have been implemented in Denmark, Norway and Sweden to improve equal access to health care across all patient groups [17–19]. Such general measures are aimed at improving survival across all cancer types, however, conceivably the benefit is greatest among patients with less advanced tumours and curable disease. This highlights the importance of early detection, where for example awareness campaigns may play an important role. In Denmark and Iceland, public awareness campaigns have been launched during the study period, for example to increase awareness of skin melanoma and related signs and symptoms.

Since the early 2000s, several important measures in cancer diagnostics and therapy have been introduced which have contributed to the survival improvements across several cancer types. More precise diagnostic methods have been implemented facilitating earlier diagnosis, improved and specialised treatment, but also to higher rates of incidental diagnoses [29]. Recent advancements in cancer therapy, including new surgical methods and systemic anti-cancer therapies, including immunotherapy, have likely also contributed to the improvements in survival of several cancer types [30–34]. As these treatments, notably immunotherapy, are now being introduced for a longer array of cancer types, the advances will likely impact on future trends.

For breast and cervical cancer, early detection by screening was available in the entire study period in all five countries, except in Denmark where breast cancer screening was first fully implemented in 2007–2009 [3]. The age ranges for breast cancer screening are different in the Nordic countries, that is 50–69 years in Denmark, Finland and Norway, 40–69 years in Iceland, and 40–74 years in Sweden (Supplementary Table S1). Screening attendance is high around 80%, except in Iceland where attendance is around 60%, which may contribute to the lower survival in Icelandic women [3]. Cervical cancer screening was introduced in the 1960s in the Nordic countries, with Iceland and Finland being the first to attain national coverage and subsequent substantial reduction in cervical cancer incidence [35]. The remaining cervical cancer cases are thus likely to be more aggressive and detected outside the screening programme, and therefore contribute to the lower cancer survival.

Screening for colorectal cancer has recently been introduced in Denmark (ages 50–74 years since 2014) and in one region in Sweden (ages 60–69 years since 2008). So far, the colorectal screening initiatives are only reflected in Danish cancer incidence trends, while no related trends are apparent yet in Sweden [2, 36]. Importantly, since screening is restricted to specific age ranges, the programs will have differential influence on age-specific survival, both through true survival benefits and through lead time bias. The lower survival in the earlier period among Danish colorectal cancer patients, have in part been attributed to differences in the proportion of patients receiving curative surgery [2].

Survival of lung cancer varied considerably between the five study countries, with Finland exhibiting lower survival and less improvement over time compared to the other Nordic countries. Although the distribution of histological subtypes of lung cancer are fairly similar across the Nordic countries, a higher proportion of ‘unknown subtype’ is registered in Finland (unpublished data). Tobacco control policies have been implemented in the Nordic countries and have likely impacted on lung cancer incidence and major lung cancer subtypes across countries [37]. Yet differences in smoking patterns, smoke-less tobacco use and cessation advice, in addition to implementation of treatment guidelines and patient pathways, may have influenced the survival differences between the countries [38].

The consistent finding of lower cancer survival among older patients highlights the need for directed efforts to improve survival in this patient group. We were not able to adjust for comorbidities or overall health status of the patients, which likely have contributed to the age differences in cancer survival. Older and frail patients are less able to endure intensive therapy, and consequently experience poorer survival due to suboptimal treatment and treatment-related mortality [39]. Also, the uptake of new treatments may be slower among older patient groups. In addition, we were not able to account for TNM stage (tumor size, lymph node involvement and distant metastases) stage or other tumour factors known to vary across age, and thus may have contributed to the differential survival among the oldest patients [40]. Despite a lower survival among the oldest patients, improvements in survival were the largest in breast cancer and skin melanoma patients above 70 years. Over-diagnosis may have contributed to these improvements, as the incidence of these cancers increased the most in the oldest population [23].

The Nordic countries have among the highest cancer survival in the world [1, 4, 41]. These improvements in cancer survival have occurred since the 1960s [1–16]. Several international cancer survival comparisons have also found general improvements in cancer survival over time across numerous cancer types and countries [41–43]. In line with our findings, those international studies have also reported on differences across age groups, with higher survival in younger age groups [40, 44–46]. For example, the SURVMARK-2 initiative, including data from Norway and Denmark 2010–2014, reported lower 5-year net survival following colorectal cancer in older than in younger ages [40]. The age differences were present across all cancer stages (I-IV), although with a stronger age gradient among patients with advanced cancer [40, 46]. Cabasag et al. reported decreasing survival by age among patients with pancreatic cancer, also after accounting for stage [44]. Araghi et al. reported large stage differences in lung cancer survival, yet with no assessment by age at diagnosis [47].

Strengths of this study include the use of population-based cancer registries in Nordic countries, which hold continuously updated data of high quality, completeness, and timeliness. The similar coding practices in the Nordic cancer registries also ensured good comparability across countries and over time. The NORDCAN database is a unique international resource with harmonised and quality-checked data including almost all new cancer cases in the Nordic countries [48]. In addition, we employed solid and validated methods for estimating RS and we only presented estimates based on a sufficient number of patients thereby avoiding large random variation.

A major limitation was lack of information on TNM stage and subtypes of cancer, which are important predictors for cancer survival. Information on TNM stage and subtypes is not yet included in the NORDCAN database due to the remaining work on quality assurance and comparability, as well as assessments of patient anonymity in the publicly available tables. Hence, we cannot exclude that stage and subtype differences across both sexes and age groups, as well as countries, explained parts of the results. Furthermore, we had no information on planned or received cancer therapy, which may have influenced the survival patterns, in particular among the elderly who often do not receive optimal treatment due to comorbidities and/or frailty. In Sweden, DCO cancers were not included, which may have biased survival among the older patients and for cancers with lower survival, for example pancreatic and lung cancers [49]. Due to small numbers in Iceland, not all measures could be estimated for all cancer sites and age groups.

## Conclusion

In this update of recent cancer survival trends in the Nordic population, we found substantial and consistent improvements in cancer survival across all age groups and selected cancer sites, and in both women and men. Likely explanations for these trends include the improvements in cancer diagnostics and cancer therapy, with earlier detection and more specialised treatment available in recent years, as well as the implementation of national care strategies and population-based screening. Despite these favourable findings, we observed a consistently poorer survival in older patients across all included cancer sites, which may be due to comorbidities and less access to recent advancements in cancer therapy. Age disparities in cancer survival thus need to be better understood.

## Supplementary Material

Have the recent advancements in cancer therapy and survival benefitted patients of all age groups across the Nordic countries? NORDCAN survival analyses 2002–2021

Have the recent advancements in cancer therapy and survival benefitted patients of all age groups across the Nordic countries? NORDCAN survival analyses 2002–2021

## Data Availability

The estimates in this paper are publicly available from the NORDCAN online database (https://nordcan.iarc.fr/en/). The data are available from the registry holder of each specific health registry under the use of appropriate ethical and legal permissions, including the GDPR. Further details and other data that support the findings of this study are available from the corresponding author upon request.
