# Supplementary material for: Have the recent advancements in cancer therapy and survival benefitted patients of all age groups across the Nordic countries? NORDCAN survival analyses 2002–2021
Source: Acta Oncol. 2024 Apr 10;63:35094. doi: 10.2340/1651-226X.2024.35094 (PMC11332520; doi:10.2340/1651-226X.2024.35094)

Supplementary material has been published as submitted. It has not been copyedited or typeset by Acta Oncologica.

Supplemental Figure S1. Age-specific cancer incidence and mortality in the Nordic countries for men and women in 2017- 2021, for all sites of cancer (except non-melanoma skin cancer).

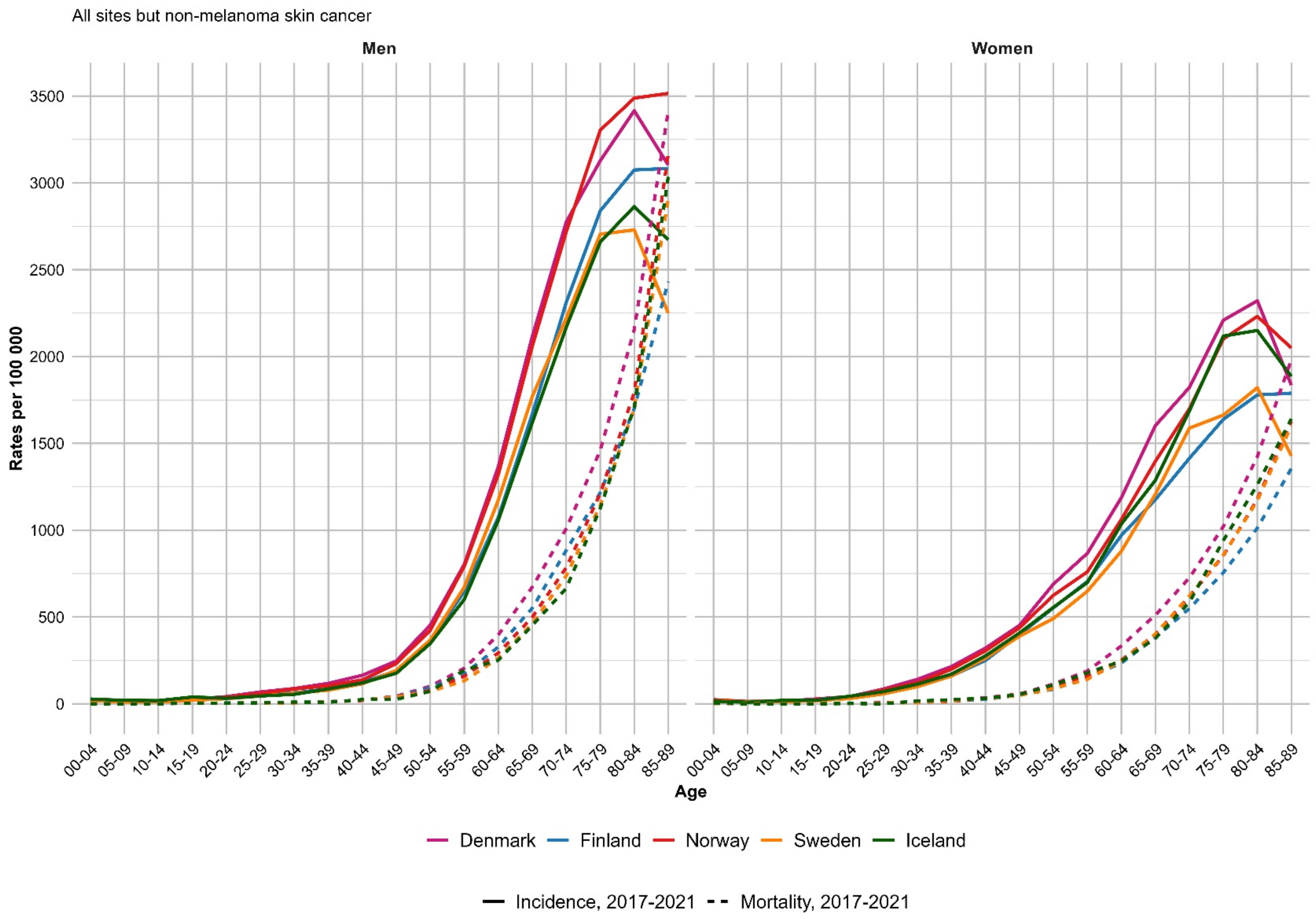

Supplemental Figure S2. Age-specific cancer incidence in the Nordic countries for men and women in 2017-2021 by major cancer sites.

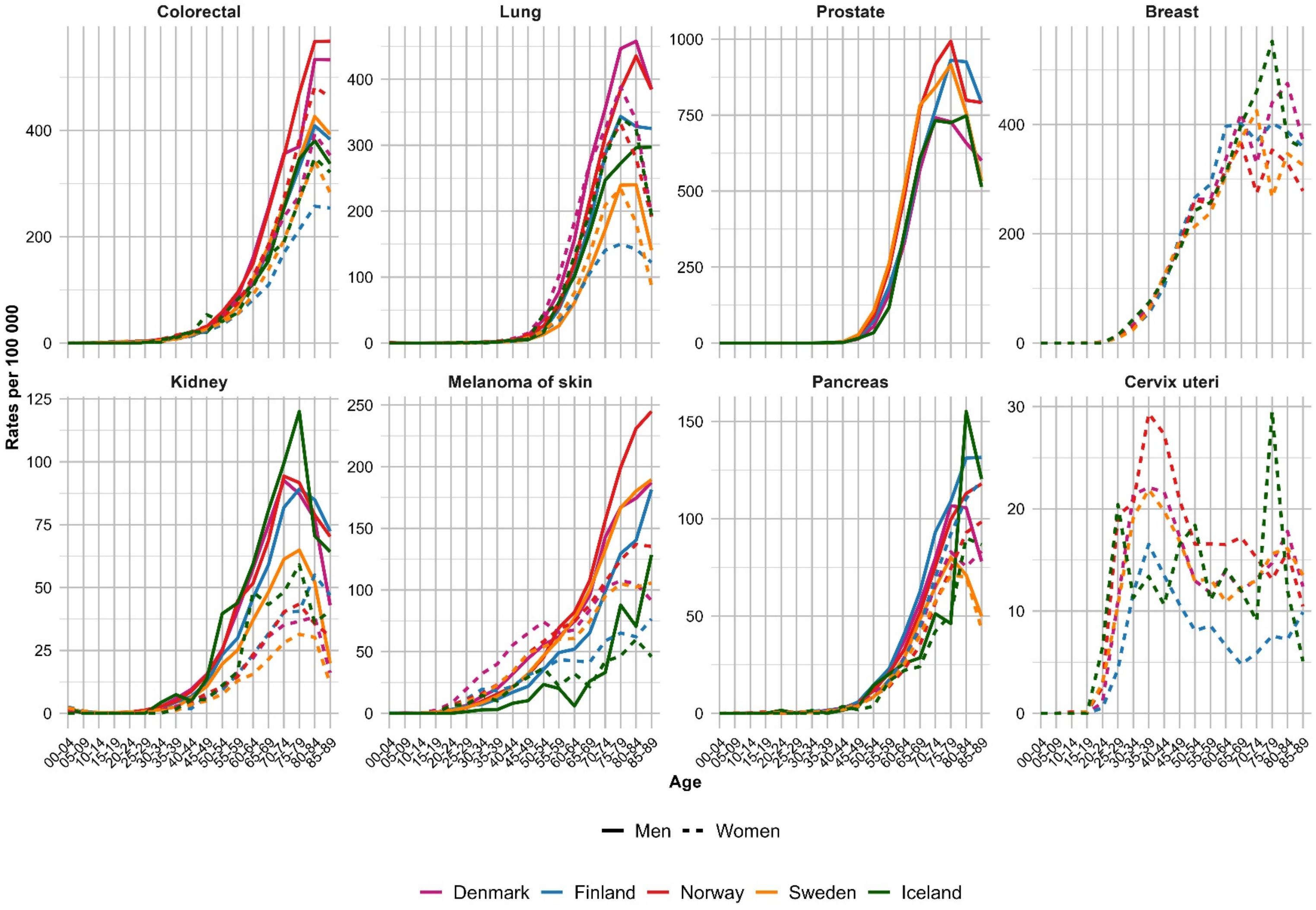

Supplemental Figure S3. Five-year relative survival across year per age, country and sex by major cancer sites. Panel (a) colorectal, (b) lung, (c) prostate, and (d) breast cancer.

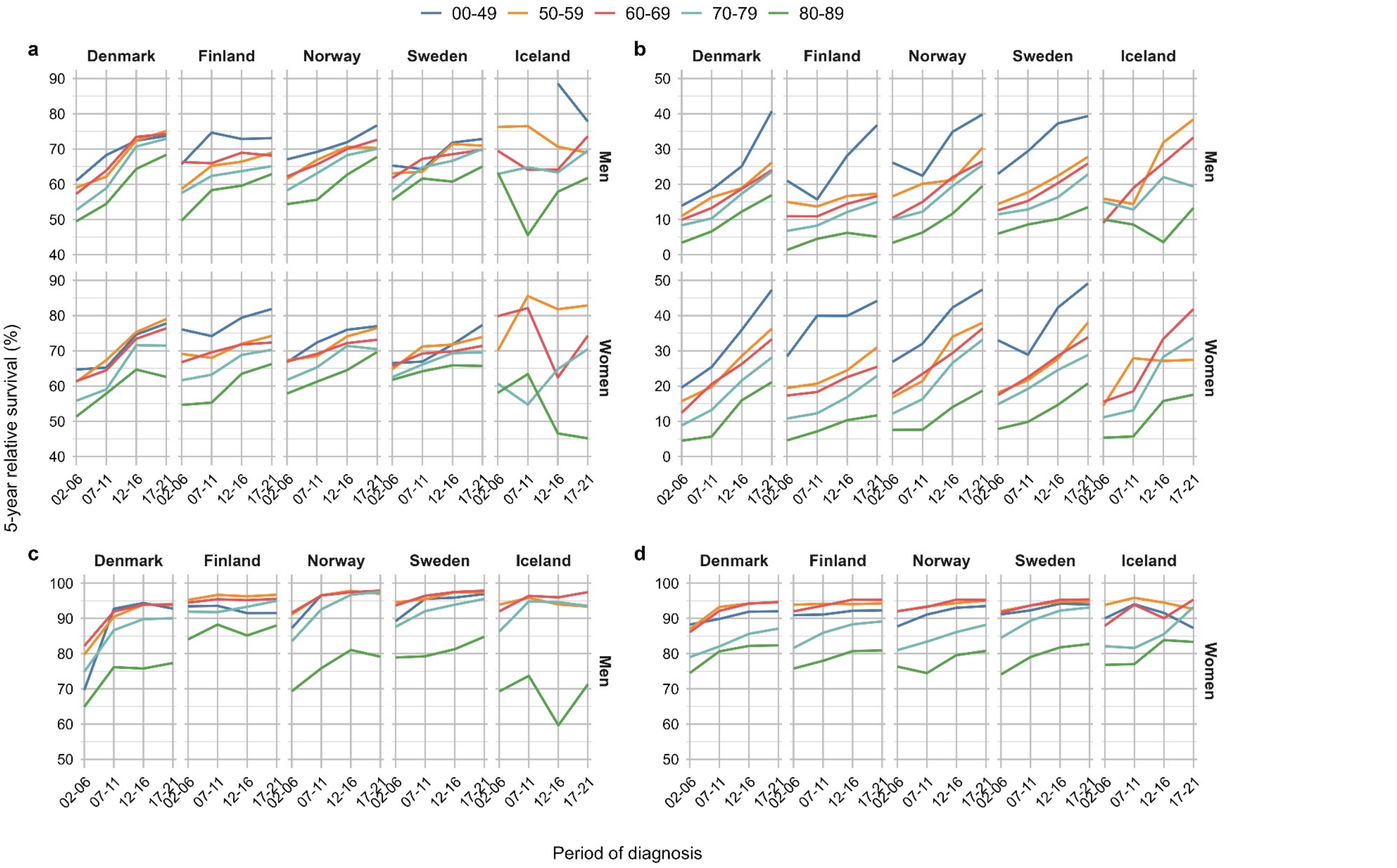

Supplemental Figure S4. Five-year relative survival across year per age, country and sex by major cancer sites. Panel (a) kidney, (b) melanoma of skin, (c) pancreas, and (d) cervix uteri.

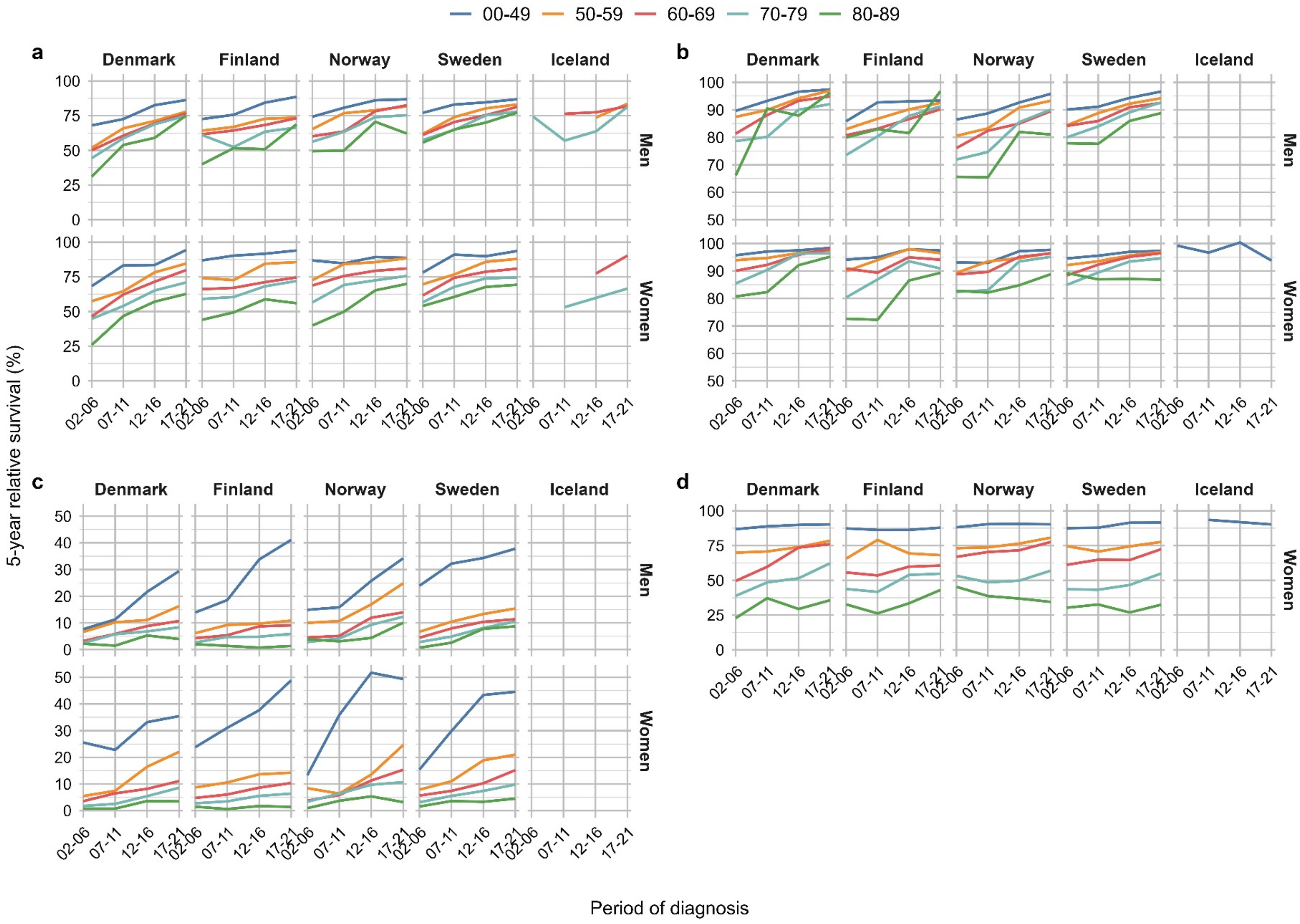

Supplement: Have the recent advancements in cancer therapy and survival benefitted patients of all age groups across the Nordic countries? NORDCAN survival analyses 2002–2021 [file AO-63-35094-s1.pdf]
