# Supplementary material for: Have the recent advancements in cancer therapy and survival benefitted patients of all age groups across the Nordic countries? NORDCAN survival analyses 2002–2021
Source: Acta Oncol. 2024 Apr 10;63:35094. doi: 10.2340/1651-226X.2024.35094 (PMC11332520; doi:10.2340/1651-226X.2024.35094)
Supplement: Have the recent advancements in cancer therapy and survival benefitted patients of all age groups across the Nordic countries? NORDCAN survival analyses 2002–2021 [file AO-63-35094-s2.pdf]

Supplementary material has been published as submitted. It has not been copyedited or typeset by Acta Oncologica.

Supplemental Table S1. Overview of cancer strategies and screening for breast, cervical and colorectal cancer in the Nordic countries.

	Denmark[5]	Faroe Islands	Finland	Iceland	Norway	Sweden
**National cancer plans[1]**	Introduced in 2000, updates 2005, 2010, 2016	First cancer plan launched in 2009, and updated 2022.	Proposal for intensifying and improving cancer treatments published 2010	First cancer plan developed 2013-2016 and accepted by Ministry of Health 2019	Implemented 1999-2003, followed by national cancer strategy 2006	Cancer strategy launched 2011 and cancer plans since 2013
**National care guidelines**	Cancer specific accelerated patient pathways established 2007-2009 and later copied in Norway and Sweden	The Danish guidelines are the main guidelines for the Faroe Islands.	Cancer specific national treatment guidelines since 1992	Treatment guidelines adapted from Nordic, European or American versions	Standardized patient assessment and treatment processes introduced 2014	Cancer specific national guidelines developed and updated by the Regional Cancer Centers since 2012
**Screening for breast cancer[2]**	Implemented in some regions in the 1990's and for the remaining 80% of women ages 50-69 in 2007-2009, now biannual for ages 50-69	Started in 2011 for ages 50-69. Plan for ages 45-74 in 2024.	Implemented gradually 1987-1991 in women ages 50-64, from 1992 organised systematically for 50-59, from 2007 gradually added women 60-69 years. Now biannual for ages 50-69.	Started in 1987 for ages 40-69, now biannual for ages 40-69 and triannual for ages 70-74	Stepwise implementation 1995-2005, now biannual for all women ages 50-69	Started in late 1980's, nationwide from 1990's for ages 50-69, now biannual for ages 40-74
**Screening for cervical cancer[3]**	Started in 1962, covered 40% of population by 1967, nationally since 1996, currently including ages 23-64	Started in 2006.	Started in 1963, essentially nationally since 1970, currently including ages 30-65	Started 1964, nationally since 1969, currently including ages 23-64	Pilot screening 1959-1977, nationally since 1995, currently including ages 25-69	Started 1967, nationally since 1977, currently including ages 23-64
**Screening for colorectal cancer[4]**	Implemented nationally since 2014-2018, currently including ages 50-74	Planned for 2024.	First programme in 2004-2016 among 60-68 years old men and women	Planning of national screening is ongoing	Implemented nationally since 2022 for age 55.	Implemented in Region Stockholm and Gotland since 2008-2009 ages 60-

			biannually in voluntary municipalities, by 2008 around 40% of the target population covered (but half screened and half controls). From 2022 national programme for 60-68 years old, gradually expanding to 56-74 years old, semi-annually.			69, will be implemented nationally in ages 60-74 years gradually 2023-2026

[1] More information on cancer plans: http://www.epaac.eu/national-cancer-plans
Denmark: http://www.epaac.eu/from_heidi_wiki/Denmark_National_Cancer_Plan_III_Danish.pdf [Danish];
http://www.epaac.eu/from_heidi_wiki/Denmark_National_Cancer_Plan_II_English.pdf
Finland: http://www.epaac.eu/from_heidi_wiki/Finland_Summary_of_NCP_English.pdf
Norway: http://www.epaac.eu/from_heidi_wiki/Norway_National_Strategy_for_Cancer_Control_2006_Norwegian.pdf [Norwegian];
http://www.epaac.eu/images/WP_10/National_cancer_Plans/Norway_National_Strategy_for_Cancer_Control_2013-2017_English.pdf
Sweden: https://cancercentrum.se/samverkan/om-oss/mal-och-inriktning/nationella-cancerstrategin/ [Swedish];
http://www.epaac.eu/from_heidi_wiki/Sweden_National_Cancer_Strategy_Summary_English.pdf

[2] Lundberg FE et al. Age-specific survival trends and life-years lost in women with breast cancer 1990-2016: the NORDCAN survival studies. Acta Oncol. 2022 Dec;61(12):1481-1489. doi: 10.1080/0284186X.2022.2156811.

[3] Partanen V-M et al. Adherence to international recommendations in the governance and organisation of Nordic cervical cancer screening programmes. Acta Oncol. 2020 Nov;59(11):1308-1315. doi: 10.1080/0284186X.2020.1821089.

[4] Njor SH et al. Three years of colorectal cancer screening in Denmark. Cancer Epidemiol. 2018 Dec:57:39-44. doi: 10.1016/j.canep.2018.09.003.

[5] More information on **Greenland**: Greenland has introduced screening for cervical cancer but experience a low participation, while screening for colorectal and breast cancer is not introduced due the high cost per premature death prevented.
https://www.sdu.dk/da/sif/rapporter/2020/evaluering_af_kraeftplan_2013_dk

Supplemental Table S2. Quality measures of incidence, 2017-2021. All sites of cancer (except non-melanoma skin cancer), all ages.

	Cases of cancer	Deaths	Morphologically verified (MV)	Death certificate only (DCO)	Mortality-to-Incidence ratio
	N	N	%	%	M:I
**Men**					
Denmark	115,773	42,820	93.9	0.5	0.4
Finland	92,414	34,880	92.5	1.4	0.4
Iceland	4,531	1,605	93.8	0.1	0.4
Norway	94,792	29,343	93.6	1.2	0.3
Sweden	167,748	60,138	97.0	N/A	0.4
**Women**					
Denmark	105,886	37,773	93.0	0.5	0.4
Finland	84,821	30,094	92.2	1.8	0.4
Iceland	4,522	1,453	94.3	0.0	0.3
Norway	81,982	25,884	93.1	1.7	0.3
Sweden	144,669	55,111	97.4	N/A	0.4

MV: The percentage of microscopically verified cases, a measure of validity.

DCO: The percentage of cases included in incidence based on information only from the death certificates. These cases are excluded from survival estimation. Sweden does not search for cases in their Cause of Death Register (N/A).

M:I: The mortality to incidence ratio, a measure of completeness.

Quality measures for 2002-2006, 2007-2011 and 2012-2016 are publicly available from NORDCAN database online.
